# Incremental prognostic value of functional impairment assessed by 6-min walking test for the prediction of mortality in heart failure

**DOI:** 10.1038/s41598-024-53817-3

**Published:** 2024-02-07

**Authors:** Domenico Scrutinio, Pietro Guida, Maria Teresa La Rovere, Laura Adelaide Dalla Vecchia, Giovanni Forni, Rosa Raimondo, Simonetta Scalvini, Andrea Passantino

**Affiliations:** 1 Istituti Clinici Scientifici Maugeri IRCCS, Institute of Bari, Via Generale Nicola Bellomo 73/75, Bari, Italy; 2 Regional General Hospital “F. Miulli”, Acquaviva Delle Fonti, Bari, Italy; 3 Istituti Clinici Scientifici Maugeri IRCCS, Institute of Montescano, Pavia, Italy; 4 Istituti Clinici Scientifici Maugeri IRCCS, Institute of Milano, Milan, Italy; 5 Istituti Clinici Scientifici Maugeri IRCCS, Institute of Pavia, Pavia, Italy; 6 Istituti Clinici Scientifici Maugeri IRCCS, Institute of Tradate, Varese, Italy; 7 Istituti Clinici Scientifici Maugeri IRCCS, Institute of Lumezzane, Brescia, Italy

**Keywords:** Cardiology, Cardiovascular diseases, Prognostic markers

## Abstract

Natriuretic peptides (NP) are recognized as the most powerful predictors of adverse outcomes in heart failure (HF). We hypothesized that a measure of functional limitation, as assessed by 6-min walking test (6MWT), would improve the accuracy of a prognostic model incorporating a NP. This was a multicenter observational retrospective study. We studied the prognostic value of severe functional impairment (SFI), defined as the inability to perform a 6MWT or a distance walked during a 6MWT < 300 m, in 1696 patients with HF admitted to cardiac rehabilitation. The primary outcome was 1-year all-cause mortality. After adjusting for the baseline multivariable risk model—including age, sex, systolic blood pressure, anemia, renal dysfunction, sodium level, and NT-proBNP—or for the MAGGIC score, SFI had an odds ratio of 2.58 (95% CI 1.72–3.88; p < 0.001) and 3.12 (95% CI 2.16–4.52; p < 0.001), respectively. Adding SFI to the baseline risk model or the MAGGIC score yielded a significant improvement in discrimination and risk classification. Our data suggest that a simple, 6MWT-derived measure of SFI is a strong predictor of death and provide incremental prognostic information over well-established risk markers in HF, including NP, and the MAGGIC score.

## Introduction

Heart failure (HF) is an increasingly prevalent clinical syndrome burdened by high mortality and morbidity rates and characterized by impaired functional capacity and quality of life^1,2^. Risk assessment is an integral part of the complex process of clinical decision-making in HF and, ideally, a crucial step to match the type and intensity of care with patient risk^3^. Many predictors of mortality have been identified in HF and used to develop prognostic models^4^. Age, sex, diabetes, New York Heart Association (NYHA) class, left ventricular ejection fraction (EF), systolic blood pressure (SBP), resting heart rate, natriuretic peptides (NP), creatinine, blood urea nitrogen, and sodium are the predictors most often incorporated in prognostic models^4^. Among these variables, NP are recognized as the most powerful predictors of short and long-term adverse outcomes in HF^5^; as such, they are recommended by current guidelines for risk assessment^5^. The degree of functional limitation has also been demonstrated to be a powerful, though underappreciated, predictor of mortality^6,7^. Nonetheless, the conjoint contribution of NPs and functional limitation to risk prediction has not been examined in any study aimed at developing a prognostic model for HF. The recently published Prospective Comparison of ARNI with ACEI to Determine Impact on Global Mortality and Morbidity in Heart Failure trial (PARADIGM-HF) predictive model for patients with chronic HF with reduced EF included as many as 27 predictors^8^. However, the Authors acknowledged the lack of a measure of functional capacity was a limitation^8^.

Cardiac rehabilitation (CR) has been regarded as an evidence-based pillar of HF management^9^. The key outcomes of CR have evolved over time to include effects on morbidity and mortality^10,11^. Meta-analyses of randomized clinical trials have shown that participation in CR is associated with reduced hospitalizations^12^. Data from large observational studies have shown an association between participation in CR and reduced risk of mortality^13–15^. Risk assessment and stratification are key features of a CR program^10,16^.

We hypothesized that a measure of functional impairment, as assessed by six-minute walking test (6MWT), would improve the accuracy of a prognostic model incorporating a NP in HF. The aim of the study was to determine the incremental prognostic value of functional limitation on the top of well-established prognostic factors, including N-terminal pro-brain natriuretic peptide (NT-proBNP), and to build a point-based risk score to predict 1-year mortality. To test this hypothesis, we studied patients with chronic HF admitted to inpatient CR.

## Methods

This was a multicenter observational retrospective study. The study population consisted of 1732 patients with chronic HF (International Classification of Diseases, Ninth Revision codes: 402.01, 402.11, 402.91, 404.01, 404.03, 404.11, 404.13, 404.91, 404.93, and 428.1/2/3/4/9) admitted to six specialized inpatient CR units of a nationwide Research Institute in the field of Rehabilitation Medicine in Italy between January 2013 and December 2016, who had available data for NT-proBNP and 6MWT at admission to CR. According to the national regulatory rules governing admissions to inpatient CR for HF in Italy, patients were admitted from acute-care hospitals just after a hospitalization for HF or from the community because of declining functional capacity and/or clinical status. Experienced physiotherapists performed a standardized 6MWT at admission to CR^17^.

### Data collection

Baseline measurements were obtained at the time of admission to inpatient CR. The data were extracted from the electronic Hospital Information System shared between the participating centers and entered into a REDCap database. All patients provided informed written consent to the use of their data in an anonymous form for scientific purposes. Any identifying information was removed from the database and replaced with an identification number. The study was undertaken as part of a companion research project exploring the association between change in 6-mine walking distance (6MWD) after CR and mortality in HF, which was approved by the Ethics Committee of Istituti Clinici Scientifici Maugeri on 27 July 2021 (approval number: 2576-CE). The research was performed in accordance with relevant guidelines and regulations. Survival status was ascertained by linkage to the national Health Information System. The patients were followed-up until death or November 30, 2019.

### Exposure variable

The exposure variable was severe functional impairment (SFI) defined as the inability to perform a 6MWT or a 6MWD < 300 m. This definition was adopted for three reasons. First, inability to perform a 6MWT was analyzed in previous studies and shown to be associated with increased mortality^18–20^. Second, severe impairment of functional capacity with inability to exercise or low 6MWD (< 300 m) is a key feature of advanced HF^21^. Third, 6MWD < 300 m is associated with increased mortality in HF^6^.

### Primary outcome

The primary outcome was 1-year all-cause mortality from admission to CR.

### Statistical analysis

Data are reported as mean and standard deviation (SD) or median with interquartile range (IQR) for continuous variables and as number and percentage for categorical variables. We used the Student’s t-test or the Mann–Whitney test to compare continuous variables and the χ^2^ test to compare categorical variables. Cumulative mortality rates were estimated using the Kaplan–Meier method. The median survival time was calculated as the time point at which the probability of survival equalized 50%. We performed univariate and multivariable logistic backward stepwise regression analysis to assess the association of each candidate covariate with the primary outcome. In order to develop a parsimonious model, the covariates with a p value < 0.05 at univariate analysis were included in the multivariable analysis^22^. For practical purposes, continuous variables were categorized. Each variable’s contribution to the model was assessed using the z-score. Adjusted associations for the primary outcome were displayed as odds ratios (OR) with 95% confidence intervals (CI). Two models were constructed. Model 1 (baseline risk model) included the following covariates: age, analyzed as per 10-year increase above 60; sex; diabetes; chronic obstructive pulmonary disease (COPD); atrial fibrillation; transfer from acute care hospitals to CR after a hospitalization for HF; NYHA class III/IV; EF < 0.40; SBP < 100 mm Hg; moderate-to-severe anemia defined as a hemoglobin concentration lower than 11 g/dL; estimated glomerular filtration rate (eGFR) categorized in 4 categories: ≥ 60 mL/min/1.73 m^2^, 45–59 mL/min/1.73 m^2^, 30–44 mL/min/1.73 m^2^, < 30 mL/min/1.73 m^2^; and NT-proBNP categorized in 4 categories; < 800 pg/mL, 800–1599 pg/mL, 1500–3199 pg/mL, ≥ 3200 pg/mL^8^. Sex was forced into multivariable modeling. These variables were selected because they were identified in previous studies as being the most consistent and strongest prognostic factors in HF^8,23^. Transfer from acute care hospitals to CR after a hospitalization for HF was considered as covariate since the first months after a hospitalization for HF are a well-known highly vulnerable period. Missing data for SBP (4.2%) were imputed as medians. Next, we evaluated the incremental prognostic value of SFI by adding SFI to model 1 (model 2). To further asses the value of added SFI, we evaluated its incremental prognostic value over the Meta-Analysis Global Group in Chronic Heart Failure (MAGGIC) integer score^24^, which was shown to have the best overall accuracy among major predictive models^25^. The MAGGIC score includes 13 variables and ranges between 0 and 52 points. The MAGGIC score was calculated by summing the individual contribution of each risk marker as described in the original publication^24^. Missing data for body mass index (2.4%) were imputed as medians. Finally, we developed a score-based prediction rule from model 2 by using an integer-based scoring system. The covariates significantly associated with the primary outcome in model 2 were used to build the risk score. Regression coefficients were converted to integers. Each coefficient was divided by the smallest coefficient and rounded to the nearest integer. Summation of points assigned for each predictor led to the prediction of mortality risk. Discrimination of the prognostic models was assessed by calculating the C statistic. We used the bootstrap resampling technique with 1000 replicates to obtain optimism-corrected C statistic for model 1 and model 2 and reported the mean results and bootstrap estimated 95% confidence intervals. Calibration was assessed by plotting predicted versus observed mortality by quintiles of predicted probability and by the Hosmer–Lemeshow statistic. To assess the incremental prognostic value of SFI, the following measures were considered: C statistic, global χ^2^, which is a measure of goodness-of-fit, the net reclassification improvement (NRI), which defines upward and downward movement among cases and non-cases, and the integrated discrimination improvement^26^. Since there are no operational risk thresholds for HF, we used the category-free NRI. Finally, we calculated sensitivity, specificity, positive predictive value (PPV), and negative predictive value (NPV) of the risk scores at the following thresholds of predicted risk: > 10%, > 20%, > 30%, > 40%, and > 50%. The optimal cutoff of predicted risk was defined by the Youden index. All analyses were conducted using STATA software, version 14 (Stata-Corp LP, College Station, Tex). We used the TRIPOD checklist when writing our report^27^.

## Results

Of the 1.732 patients, 36 (2.1%) were lost to follow-up. Table 1 displays the baseline characteristics of the 1696 patients analyzed in the study. Mean age was 67.9 years and 26.9% were females. The percentages of patients with reduced EF treated with beta-blockers, renin-angiotensin system inhibitors, or their association were as high as 94.0%, 90.3%, and 84.8%, respectively.

**Table 1:** Baseline characteristics.

Variables	Number of observations	Mean (SD) or N (%)
Demographics		
Age (years), mean (SD)	1696	67.9 (12.8)
Age > 70 years, N (%)	1696	794 (46.8)
Females, N (%)	1696	456 (26.9)
Body mass index, mean (SD)	1655	27.1 (6.1)
Current smokers, N (%)	1696	277 (16.3)
Etiology	1417	
Ischemic heart disease, N (%)		707 (49.9)
Dilated cardiomyopathy, N (%)		490 (31.6)
Hypertensive, N (%)		112 (7.9)
Valvular disease*, N (%)		92 (6.5)
Others, N (%)		16 (1.1)
Comorbidities		
Obesity (body mass index ≥ 30), N (%)	1655	432 (25.5)
Hypertension, N (%)	1696	810 (47.8)
Diabetes mellitus, N (%)	1696	510 (30.1)
Chronic obstructive pulmonary disease, N (%)	1696	346 (20.4)
Moderate-to-severe anemia (hemoglobin < 11 g/dL), N (%)	1696	320 (18.9)
Atrial fibrillation, N (%)	1696	630 (37.1)
Clinical findings		
Transferred from acute care hospitals after a hospitalization for HF, N (%)	1696	698 (41.2)
NYHA III/IV class, N (%)	1696	945 (55.7)/99 (5.8)
ICD, N (%)	1696	630 (37.1)
ICD in patients with reduced EF, N (%)	1696	564 (47.5)
Systolic blood pressure (mm Hg), mean (SD)	1625	111.8 (16.9)
Systolic blood pressure < 100 mm Hg, N (%)		287 (17.7)
Left ventricular ejection fraction, mean (SD)	1696	0.36 (0.13)
Left ventricular ejection fraction < 0.40, N (%)		1134 (66.9)
Inability to perform a 6MWT or 6MWD < 300 m at admission, N (%)	1696	952 (56.1)
Inability to walk, N (%)		400 (23.6)
6MWD < 300 m, N (%)		552 (32.5)
Laboratory findings		
Hemoglobin (g/dL), mean (SD)	1696	12.8 (2.0)
Creatinine (mg/dL), (mean (SD)	1696	1.39 (0.6)
eGFR (mL/min/1.73 m^2^), mean (SD)	1696	56.4 (23.8)
eGFR < 60 mL/min/1.73 m^2^, N (%)	1696	996 (58.7)
45–59 mL/min/1.73 m^2^, N (%)		411 (24.2)
30–44 mL/min/1.73 m^2^, N (%)		384 (22.6)
< 30 mL/min/1.73 m^2^, (N%)		201 (11.8)
Sodium < 136 mEq/L, N (%)	1696	302 (17.8)
NT-proBNP (pg/mL), median (IQR)	1696	2125 (836–4473)
< 800 pg/mL, N (%)		402 (23.7)
800–1599 pg/mL, N (%)		308 (18.2)
1600–3199 pg/mL, N (%)		382 (22.5)
≥ 3200 pg/mL, N (%)		604 (35.6)
Treatments in patients with EF ≤ 0.40**	1171	
Beta-blockers, N (%)		1101 (94.0)
RAAS-Is, N (%)		1057 (90.3)
Beta-blockers plus RAAS-Is, N (%)		994 (84.8)

*eGFR* estimated glomerular filtration rate, *ICD* implantable cardioverter defibrillator, *NYHA* New York Heart Association, *N* number of patients, *6MWT* 6-min walking test, *6MWD* 6-min walking distance, *SD* standard deviation.

*Most patients with valvular disease had previously been submitted to valve replacement.

**Discharged alive from cardiac rehabilitation.

Nine hundred fifty-two (56.1%) patients presented with SFI. Compared with non-SFI patients, those with SFI were older and more often females; had a higher comorbidity burden; more often had been transferred to CR from acute care after a hospitalization for HF; had higher EF, more prevalent and severe renal dysfunction, and higher NT-proBNP levels; and more often had low sodium levels (Supplementary table S1). One-year cumulative survival rate was 0.95 (95% 0.93–0.96) for non-SFI patients and 0.77 (95% CI 0.75–0.80) (p < 0.001) for patients with SFI.

### Baseline risk model (model 1)

The median follow-up was 3.58 (IQR 1.69–5.17) years. Two hundred fifty-six (15.1%) patients died within 1 year from admission to CR. There were 21 events for each candidate variable. At multivariable analysis, age, sex, transfer from acute care to CR after a hospitalization for HF, NYHA III/IV class, SBP < 100 mm Hg, moderate-to-severe anemia, eGFR < 30 mL/min/1.73 m^2^, sodium < 136 mEq/L, and NT-proBNP were identified as predictors of 1-year mortality (Model 1) (Table 2). The model had an optimism-corrected C statistic of 0.802 (95% CI 0.774–0.828) and was well calibrated (Hosmer–Lemeshow statistic 7.68; p = 0.464).

**Table 2:** Predictors of 1-year mortality.

Predictors	Model 1		Model 2			
	OR (95% CI)	p value	β coefficient	z score	OR (95% CI)	p value
Age	1.51 (1.27–1.80)	< 0.001	0.283	3.09	1.33 (1.11–1.59)	0.002
Male sex	1.90 (1.34–2.70)	< 0.001	0.735	4.08	2.09 (1.47–2.97)	< 0.001
Transfer from acute care after a hospitalization for HF	1.50 (1.10–2.04)	0.011	0.282	1.75	1.33 (0.97–1.82)	0.080
NYHA III/IV	1.51 (1.08–2.13)	0.017	0.327	1.86	1.39 (0.98–1.96)	0.063
Moderate-to-severe anemia (hemoglobin < 11 g/dL)	1.87 (1.35–2.6)	< 0.001	0.552	3.26	1.74 (1.25–2.42)	0.001
Systolic blood pressure < 100 mm Hg	1.67 (1.16–2.39)	0.005	0.547	2.95	1.73 (1.20–2.49)	0.003
eGFR < 30 mL/min/1.73 m^2^	1.93 (1.33–2.81)	0.001	0.629	3.26	1.86 (1.28–2.70)	0.001
Sodium < 136 mEq/L	1.62 (1.13–2.31)	0.008	0.435	2.37	1.54 (1.08–2.21)	0.018
NT-proBNP 800–1599 pg/mL	2.21 (1.06–4.63)	0.035	0.753	1.99	2.12 (1.01–4.46)	0.047
NT-proBNP 1600–3199 pg/mL	3.13 (1.57–6.25)	0.001	1.107	3.13	3.02 (1.51–6.05)	0.002
NT-proBNP ≥ 3200 pg/mL	7.11 (3.72–13.6)	< 0.001	1.816	5.46	6.15 (3.21–11.8)	< 0.001
Inability to perform a 6MWT or 6MWD < 300 m	–		0.949	4.57	2.58 (1.72–3.88)	< 0.001

*eGFR* estimated glomerular filtration rate, *OR* odds ratio, *CI* confidence intervals, *6MWT* 6-min walking test, *6MWD* 6-min walking distance.

### Incremental prognostic value of SFI over model 1

The unadjusted OR for SFI was 5.16 (95% CI 3.62–7.35). After adjusting for model 1, SFI had an OR of 2.58 (95% CI 1.72–3.88; p < 0.001) (Table 2). When the analysis was restricted to the patients able to perform a 6MWT, low 6MWD (< 300 m) remained independently associated with primary outcome (OR 2.41 [95% CI 1.53–3.79]; p < 0.001). When body mass index, modeled as per 5-unit increase, was forced into multivariable modeling, the OR for SFI remained nearly unchanged (2.76 [95% CI 1.82–4.21]; p < 0.001).

Model 2—that is, model 1 plus SFI—had an optimism-corrected C statistic of 0.814 (95% CI 0.787–0.840) and was well calibrated (Hosmer–Lemeshow statistic 13.32; p = 0.101). NT-proBNP ≥ 3200 pg/mL (z-score: 5.46) and SFI (z-score: 4.57) provided the highest contribution to the model (Table 2). Adding SFI to model 1 yielded a statistically significant increase in C-statistic, global χ^2^, and the proportion of patients with events reclassified upwards (Table 3).

**Table 3:** Incremental prognostic value of severe functional impairment.

	Model 1	Model 2	p value
C statistic (95% CI)	0.802 (0.774–0.828)	0.814 (0.787 to 0.840)	0.018
Global χ^2^	259.89	282.38	< 0.001
Category-free-NRI (95% CI)	–	0.417 (0.284 to 0.550)	< 0.001
Category-free NRI for events (95% CI)	–	0.352 (0.219 to 0.485)	< 0.001
Category-free-NRI for non-events (95% CI)	–	0.065 (− 0.068 to 0.198)	0.336
IDI (95% CI)	–	0.013 (0.007 to 0.019)	< 0.001
IDI for events (95% CI)	–	0.011 (0.005 to 0.017)	< 0.001
IDI for non-events (95% CI)	–	0.002 (0.000 to 0.004)	0.066

*NRI* net reclassification improvement, *IDI* integrated discrimination improvement.

### Incremental prognostic value of SFI over the MAGGIC score

**Figure 1:**
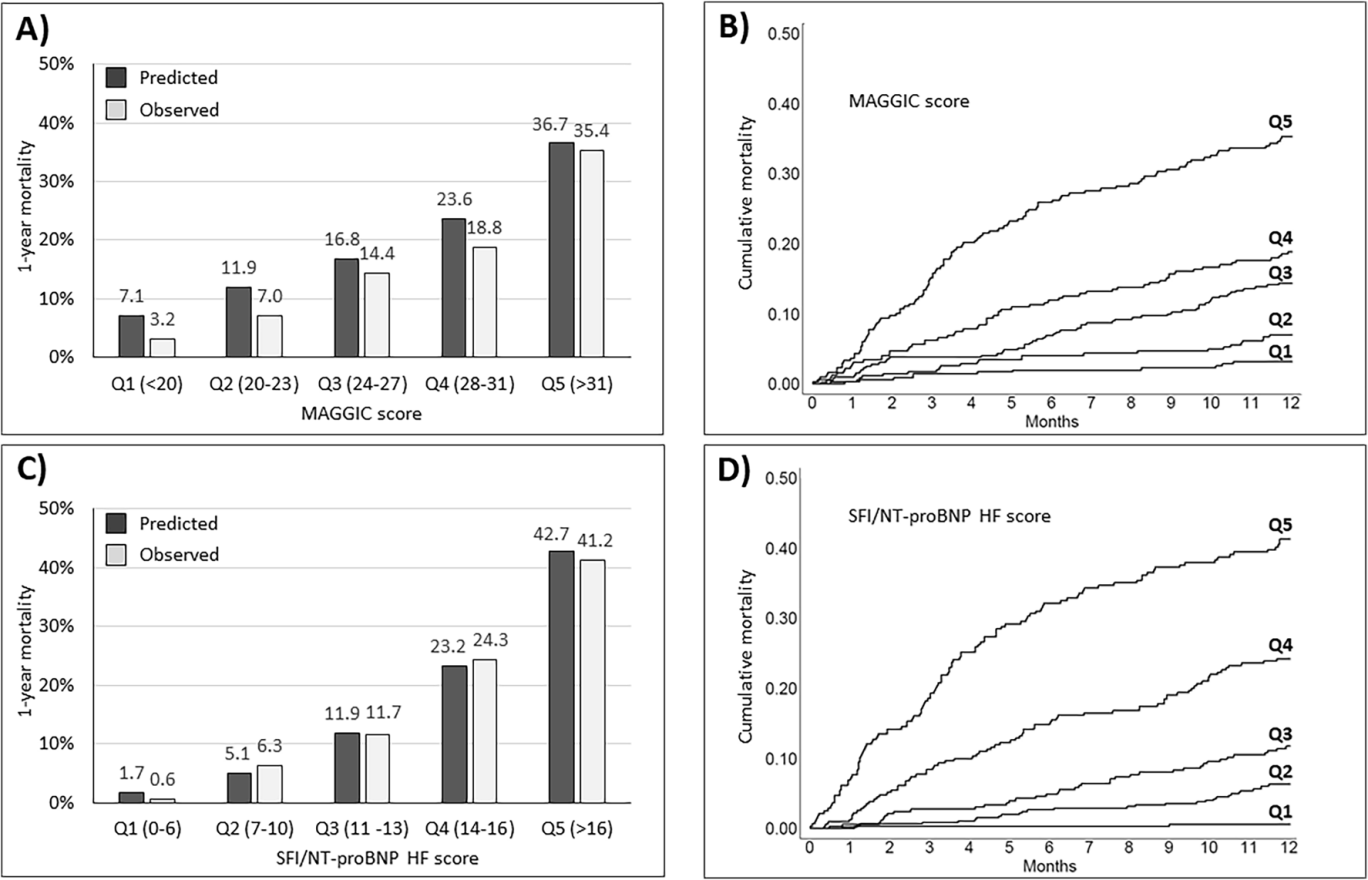
(**A**) MAGGIC score-predicted vs. observed 1-year mortality by quintiles of risk score. (**B**) cumulative mortality rates by quintiles of the MAGGIC score. (**C**) SFI/NT-proBNP HF score-predicted vs. observed 1-year mortality by quintiles of risk score. (**D**) Cumulative mortality rates by quintiles of the SFI/NT-proBNP score.

The median MAGGIC score was 25 (IQR 21–30). The associated OR per 1-point increase in MAGGIC score was 1.11 (95% CI 1.08–1.14; p < 0.001). The MAGGIC score had a C statistic of 0.717 (95% CI 0.685–0.750). Figure 1 displays predicted vs observed 1-year mortality by quintiles of MAGGIC score (panel A) and cumulative mortality curves across quintiles of the MAGGIC score (panel B). The MAGGIC score overestimated mortality risk for patients classified into the 1st to the 4th quintiles, while predicted mortality (36.7%) closely matched observed mortality (35.3%) in the 5th quintile.

After adjusting for the MAGGIC score, the ORs for SFI was 3.12 (95% CI 2.16–4.52; p < 0.001). After further adjustment for NT-proBNP, SFI remained independently associated with 1-year mortality (OR 2.62 [95% CI 1.80–3.82]; p < 0.001). Adding SFI to the MAGGIC score yielded a large, statistically significant improvement in discrimination and risk classification (Table 4), which further improved when even NT-proBNP was added to the model (Table 4).

**Table 4:** Incremental prognostic value of severe functional impairment and NT-proBNP over the MAGGIC score.

	MAGGIC score	MAGGIC score plus SFI	p value	MAGGIC Score plus SFI and NT-proBNP	p value
C statistic (95% CI)	0.717 (0.685–0.750)	0.754 (0.722 to 0.785)	< 0.001	0.791 (0.761–0.817)	< 0.001
Global χ^2^	161.77	203.63	< 0.001	256.45	< 0.001
Free-NRI (95% CI)	–	0.595 (0.463 to 0.728)	0.838	0.638 (0.505–0.771)	< 0.001
Free-NRI for events (95% CI)	–	0.609 (0.476 to 0.742)	< 0.001	0.352 (0.219–0.485)	< 0.001
Free-NRI for non-events (95% CI)	–	− 0.014 (− 0.147 to 0.119)	< 0.001	0.286 (0.153–0.419)	< 0.001
IDI (95% CI)	–	0.023 (0.016 to 0.030)	< 0.001	0.055 (0.044–0.067)	< 0.001
IDI for events (95% CI)	–	0.020 (0.014 to 0.026)	< 0.001	0.047 (0.036–0.058)	< 0.001
IDI for non-events (95% CI)	–	0.003 (0.001 to 0.006)	0.017	0.008 (0.004–0.013)	< 0.001

*NRI* net reclassification improvement, *IDI* integrated discrimination improvement.

### The severe functional impairment/NT-proBNP (SFI/NT-proBNP) risk score

**Figure 2:**
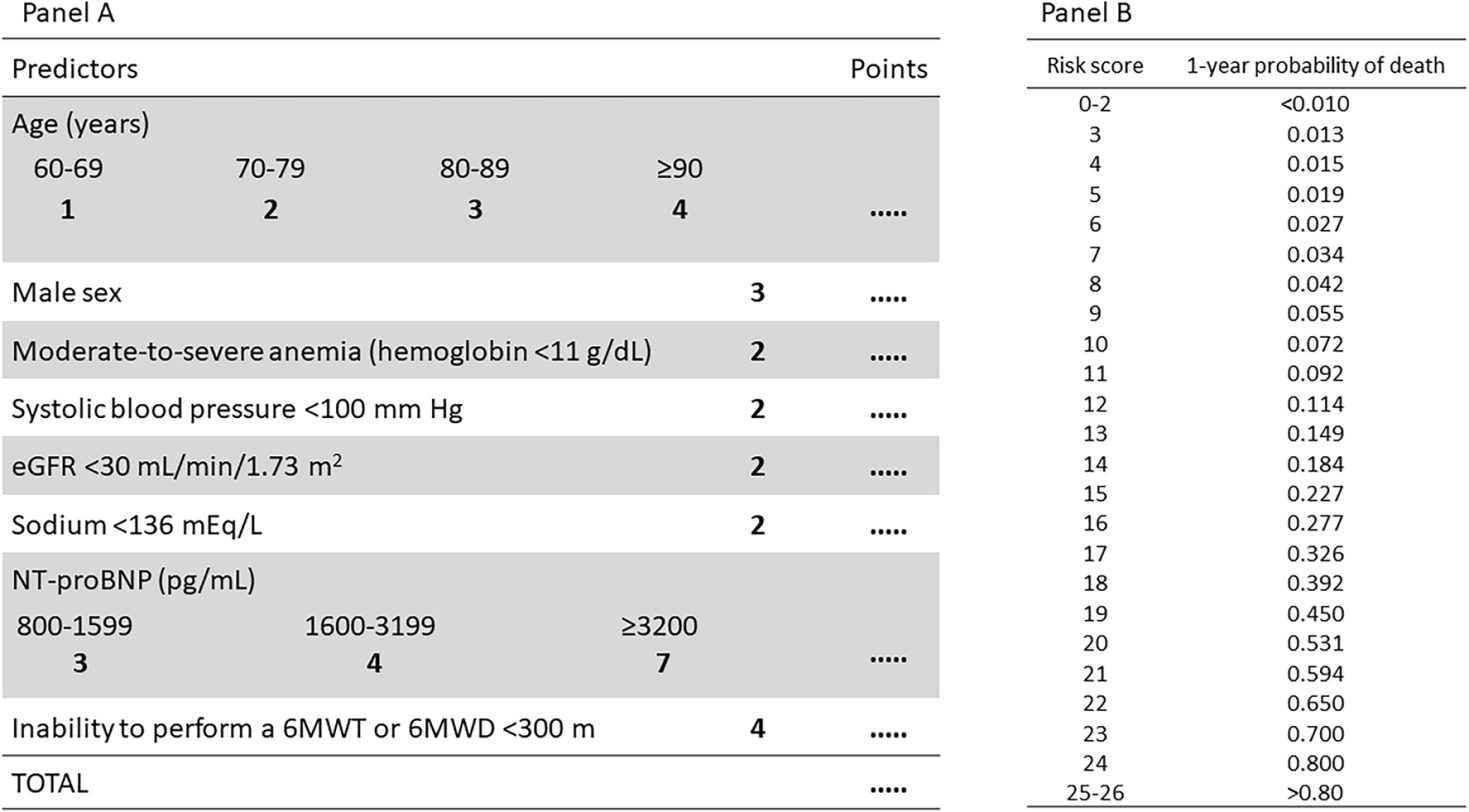
(**A**) Chart to calculate the Severe Functional Impairment/NT-proBNP HF risk score. (**B**) Predicted probabilities of 1-year mortality for each value of the risk score. *6MWT* 6-min walking test, *6MWD* 6-min walking distance.

The covariates significantly associated with the primary outcome in model 2 (age, sex, SBP < 100 mm Hg, moderate-to-severe anemia, eGFR, sodium, NT-proBNP, and SFI) were used to build a point-based risk score, termed the SFI/NT-proBNP HF risk score (Fig. 2A). The possible point totals for each patient ranged from 0 to 26. Figure 2B displays predicted probabilities of 1-year mortality for each value of the risk score. For instance, a score of 10 or 20 points predicted a 1-year mortality of 7.2% or 53.1%, respectively. Supplementary Fig. S1 displays the distribution of the patients across the risk score. Supplementary Fig. S2 displays the prevalence of individual predictors across quintiles of the risk score. The prevalence of both components of SFI—inability to perform a 6MWT and 6MWD < 300 m—progressively increased across quintiles of increased risk; the two components were equally represented in the highest risk quintile.

The median score was 11 (IQR 7–15). The associated OR per 1-point increase in score was 1.30 (95% CI 1.26–1.36; p < 0.001). The risk score had a C statistic of 0.805 (95% CI 0.778–0.832). Figure 1C displays predicted vs observed 1-year mortality by quintiles of score; Fig. 1D displays cumulative mortality rates across quintiles of the SFI/NT-proBNP score. Observed mortalities matched predicted mortalities. The median survival was 5.4 years for the patients classified into the 3rd quintile, 2.6 years for those classified into the 4th quintile, and 1.3 years for those classified into the 5th quintile.

### Sensitivity, specificity, PPV, and NPV of the risk scores

Table 5 displays sensitivity, specificity, PPV, and NPV of the SFI/NT-proBNP and the MAGGIC risk scores at the thresholds of 10%, 20%, 30%, 40%, and 50% predicted risk. Based on the Youden index, a predicted risk > 20% was the optimal cutoff for both the MAGGIC score and the SFI/NT-proBNP risk score. 36.3% of the patients were predicted to have a risk > 20% by the MAGGIC score and 28.1% by the SFI/NT-proBNP HF score. Using the risk threshold of > 20%, the SFI/NT-proBNP HF and the MAGGIC scores correctly identified 67.6% and 64.4% of the patients who actually died within 1 year, respectively. Of the patients surviving one year, 21.1% and 31.3% were predicted to have a 1-year probability of survival < 80% by the SFI/NT-proBNP HF score and the MAGGIC score, respectively. At the optimal risk threshold of > 20%, the MAGGIC and the SFI/NT-proBNP HF risk scores had a PPV of 26.8% and 36.3%, respectively. The corresponding NPVs were well above 90%.

**Table 5:** Sensitivity, specificity, positive predictive value, and negative predictive value of increasing thresholds of predicted risk.

Predicted probality of death	Risk scores	N. at risk (%)	Score	Predicted risk (%)	Observed mortality (%)	Sensitivity	Specificity	Youden index	PPV	NPV
> 10%	MAGGIC score	1347 (79.4)	> 19	21.5	18.2	95.7	23.5	0.192	18.2	96.9
	SFI/NT-proBNP score	807 (47.6)	> 11	27.0	26.6	84.8	59.0	0.438	26.9	95.6
> 20%	MAGGIC score	616 (36.3)	> 27	29.9	26.8	64.4	68.7	0.331	26.8	91.6
	SFI/NT-proBNP score	477 (28.1)	> 14	35.5	36.3	67.6	78.9	0.464	36.3	93.2
> 30%	MAGGIC score	227 (13.4)	> 32	39.1	36.1	32.0	89.9	0.219	36.1	88.2
	SFI/NT-proBNP score	274 (16.2)	> 16	42.7	41.2	44.1	88.8	0.329	42.8	89.9
> 40%	MAGGIC score	64 (3.8)	> 37	49.8	54.7	13.7	98.0	0.117	54.7	86.5
	SFI/NT-proBNP score	118 (7.0)	> 18	52.2	51.7	23.8	96.0	0.198	51.7	87.6
> 50%	MAGGIC score	21 (1.2)	> 39	59.8	71.4	5.9	99.6	0.055	71.4	85.6
	SFI/NT-proBNP score	68 (4.0)	> 19	57.5	54.4	14.5	97.9	0.124	54.4	86.6

*PPV* positive predictive value, *NPV* negative predictive value.

### Effect of CR in patients with SFI

Of the 952 patients presenting with SFI at admission to CR, 24 (2.5%) did not perform a 6MWT at discharge because of death in the inpatient rehabilitation facility or transfer from CR to acute care and 44 (4.6%) had missing data for 6MWT at discharge. Of the remaining 884 patients, 237 (26.8%) achieved an increase in 6MWD to 300 m or more after CR (mean increase: 158 ± 115 m) and 647 did not. The proportion of patients who achieved an improvement in 6MWD ≥ 300 m progressively decreased across quintiles of increasing risk (Supplementary Fig. S3). Improvement in 6MWD to ≥ 300 m was associated with an unadjusted OR for 1-year mortality of 0.30 (95% CI 0.19–0.48; p < 0.001). The improvement in survival was more pronounced in high risk patients (Supplementary Fig. S4). After adjusting for the SFI/NT-proBNP HF score, the OR of improvement in 6MWD to ≥ 300 m for 1-year mortality was 0.37 (95% CI 0.23–0.61; p < 0.001).

## Discussion

Three major findings emerged from this study. First, a simple, 6MWT-derived measure of SFI was strongly associated with 1-year mortality. Second, SFI provided incremental prognostic information beyond a model including powerful markers of mortality risk in HF and beyond the MAGGIC score. Third, the point-based SFI/NT-proBNP HF scoring system, which combines demographic, clinical, biochemical, and functional indices, demonstrated good performance in predicting 1-year mortality.

We used the 6MWT to assess functional capacity. Undisputedly, cardiopulmonary exercise testing (CPET) is the gold standard method to assess the prognostic value of functional capacity in HF^7^. However, some aspects of this important technique, such as the “need for additional expensive equipment and personnel who are proficient in the administration and interpretation of the test”, limit its broad clinical application^28^. In addition, a non-negligible proportion of patients do not achieve a peak respiratory exchange ratio of ≥ 1.05, making the interpretation of test results more difficult, or do not have interpretable CPET results^29^. Moreover, severely symptomatic patients, who are accustomed to limiting their daily physical activities because of early onset of dyspnea or fatigue during daily life, may be unwilling to perform a maximal symptom-limited exercise test^7^. The standardized 6MWT is a safe, inexpensive, reliable, and reproducible tool to assess functional capacity^5^. In addition, it has been used to evaluate treatment efficacy and to predict prognosis in HF^6,30,31^. Data from the HF-ACTION trial also suggest that the 6MWT provides prognostic utility comparable to that of CPET in HF patients with reduced EF^31^. Finally, a 6MWT may represent maximal effort in severely impaired patients^32^. Consistently, a recent study showed that, in patients with severe exercise limitation, the peak VO_2_ measured by a portable device allowing breath-by-breath measurement of cardiorespiratory parameters during a standard 6MWT is similar to -or even higher than- that reached in CPET^33^.

A particular strength of our model was the inclusion of both a NP and a measure of functional impairment in the predictive model. Our data demonstrate that a simple 6MWT-derived measure of SFI, that is, inability to perform a 6MWT or a distance covered during a 6MWT < 300 m, had a strong predictive value for the primary end point. Ranked by z score, SFI was the second most powerful predictor of 1-year mortality after high NT-proBNP levels and was associated with 2.6-fold increased odds of 1-year mortality. It is worth noting that when the analysis was restricted to the patients able to perform a 6MWT, a low 6MWD (< 300 m) remained independently associated with the primary outcome, with 2.4-fold increased odds of death. Moreover, the prevalence of the two components of SFI—that is, inability to perform a 6MWD and low 6MWD (< 300 m)—progressively increased across quintiles of increasing risk, with inability to perform a 6MWT and low 6MWD being equally represented in the highest risk quintile. These findings confirm and strengthen the results of Bittner et al. who found a 6MWD < 300 min to be strongly associated with mortality risk^6^. SFI added valuable prognostic information to the baseline risk model, leading to significant improvement in model fit. Adding SFI to the baseline risk model also resulted in more accurate risk classification of the patients who experienced the primary outcome; 35.2% of these patients were correctly reclassified upwards. We also evaluated the MAGGIC score in our population. Its discrimination (0.717), as well as the increase in odds of death for each point increase in score (0.11), was in the range of values reported in previous validation studies^24,34^. However, the MAGGIC score tended to overestimate mortality risk, although predicted mortality closely matched observed mortality in the highest quintile of risk. When SFI was added to the MAGGIC score, discrimination significantly improved to 0.754; an even larger improvement to 0.791 was observed when both SFI and NT-proBNP were added. The addition of SFI to the MAGGIC score also improved risk classification; the majority (60.9%) of the patients who experienced the primary outcome were correctly reclassified upwards. In contrast the addition of SFI to either the baseline risk model did not improve risk reclassification of survivors. Collectively, these findings support the use of 6MWT for risk stratification in HF and suggest that combining SFI with established risk markers, including NT-proBNP that is recognized as the strongest prognostic marker, can significantly improve the accuracy of risk prediction and provide a meaningful improvement in the identification of the patients at risk of death.

Based on these data, we developed a new multi-parametric scoring system, the SFI/NT-proBNP HF risk score, for prediction of 1-year mortality in patients with chronic HF. To our knowledge, this is the first scoring system combining demographic, clinical, biochemical (including NT-proBNP), and functional parameters to assess prognosis in HF. The SFI/NT-proBNP HF risk score had a C statistic of 0.805, indicating good to excellent discrimination, and was well-calibrated. For ach point increase in risk score, the odds of death increased by 1.30 times. The risk score allowed identifying a very marked gradient in risk, with 1-year mortality ranging from < 1% in the lowest quintile to 41.2% in the highest quintile. The long-follow-up allowed us estimate the median survival. Very impressively, 50% of the patients classified in the quintile of highest predicted risk (5th quintile) died within 1.3 years, while the time point at which survival equalized 50% was 2.6 years in the 4th and 5.4 years in the 3rd quintile. Sensitivity, specificity, PPV, and NPV of risk models are more informative about clinical value than discrimination and calibration. At the optimal threshold of > 20% predicted risk of death, the SFI/NT-proBNP HF score performed fairly better than the MAGGIC score. The SFI/NT-proBNP HF score and the MAGGIC score correctly identified 67.6% and 64.4% of the patients who actually died, respectively, while approximately one fifth of the patients surviving one year were predicted to have a 1-year probability of survival < 80% by the SFI/NT-proBNP score compared with one third by the MAGGIC score. However, the PPV—that is, the probability that a patient will die when classified as being at high risk—of the SFI/NT-proBNP HF score, was 36.3%, implying that the proportion of false positives exceeded that of true positives. The PPV for the MAGGIC score was lower (26.8%). Raising the threshold to > 30% resulted in a modest improvement in PPV, but at the cost of a marked decrease in sensitivity. These findings indicate that, though the risk scores performed well for stratifying patients into clinically meaningful risk groups, their use for predicting prognosis at the individual level left uncertainties. This phenomenon was already observed for major risk models in the setting of acute and chronic HF^35,36^.

Ideally, the prognostic information afforded by risk models should translate into improved clinical decision-making and management, and, eventually, into improved outcomes of HF; otherwise, risk prediction by even the most accurate predictive model would be futile in the clinical setting. Unfortunately, there is a dearth of knowledge about this topic and research efforts in developing prognostic models for HF have not generated “actionable knowledge that improves management and outcomes”^37^. Two recently published randomized clinical trials performed in the acute care setting provided somewhat conflicting results. The Risk Evaluation and its Impact on Clinical Decision Making and Outcomes in Heart Failure (REVEAL-HF) study, investigated whether provision of 1-year mortality estimates during HF hospitalization affects outcomes in patients hospitalized for HF^38^. There was no evidence that information about risk affected the rate of 30-day hospital readmissions and mortality, prescription of HF medications at discharge, or 1-year mortality. The Authors suggested “algorithm aversion” as a likely explanation for the negative results^38^. The recently published Comparison of Outcomes and Access to Care for Heart Failure (COACH) trial enrolled patients seeking care for HF at the Emergency Department^39^. The use of a clinical algorithm to predict risk of death within 7 days and within 30 days, combined with the provision of standardized transitional care, led to a statistically significant 12% lower risk of all-cause death or hospitalization for cardiovascular causes within 30 days and 5% within 20 months. However, 20-month mortality was unaffected (10.9% in the control group and 10.1% in the intervention group)^39^.

Targeting high-risk patients is a valid option for addressing the clinical application of risk scores to improve management and outcomes for several reasons. First, a risk-treatment mismatch, “where treatment rates among eligible patients are inversely associated with risk of mortality”, has been consistently demonstrated in HF^40–43^, suggesting that in high-risk patients there is room for improving pharmacotherapy. Second, there is evidence that the higher the baseline risk, the higher is the absolute risk reduction achieved with recommended treatments^44–47^. Consistent with this concept, in the present study, the absolute gain in survival associated with improved functional capacity after CR among patients presenting with SFI progressively increased with increasing predicted risk. Third, according to Pfeiffer and Gail^48^, the concept of “concentration of risk”—that is, the degree of concentration of the cases in the high-risk subgroup and the proportion of patients needed to follow in order that a proportion p of those destined to become cases will be followed”—is directly relevant to the clinical usefulness of predictive models. In the present study, approximately two thirds of the observed deaths across all participants were concentrated in the high-risk subgroup defined by a > 20% predicted risk, regardless of the score used to predict risk. However, the proportion of patients with a predicted risk > 20%—that is, the proportion of patients needed to follow—was significantly lower using the SFI/NT-proBNP score than using the MAGGIC score (28.1% vs 36.3%). Finally, targeting high-risk patients would prevent the dilution of the potential benefit due to the inclusion of low-risk patients. Combining the provision of a predicted risk > 20% with standardized, guideline-directed actions to be taken to improve management—such as, monitoring of laboratory parameters, closer follow-up, prescription/up-titration of recommended HF medical therapy, multi-specialty management of non-cardiac comorbidities, consideration for cardiac resynchronization therapy, personalized CR, or, eventually, referral to an advanced HF center^2,49^ might lead to improved prognosis. Fonarow et al. estimated that each 10% improvement in composite care was associated with 13% lower odds of 24-month mortality in outpatients with reduced EF and chronic HF or post-myocardial infarction^50^. Ultimately, however, the choice of any absolute risk threshold for interventions will depend on the expected risk–benefit ratio and the sustainability of the expected increased costs.

Compared with non-SFI patients, those presenting with SFI were 11 years older. Functional decline is inherent to aging. Fleg et al. demonstrated that longitudinal rate of functional decline in healthy adults is not linear, but accelerates dramatically as age advances^51^. An important implication of this observation is that older persons are more vulnerable to developing functional dependence for activities of daily living than younger persons when multimorbidity or disease-related deficits are superimposed and when hospitalizations occur^51^. In addition, patients presenting with SFI had higher comorbidity burden, more often had directly been transferred to CR from acute care hospitals after a hospitalization for HF—that is, when the risk of adverse clinical events is highest, had higher NT-proBNP levels, and had poorer prognosis, thus representing a highly challenging population in the CR setting. Approximately one in four patients presenting with SFI derived a substantial functional benefit from CR, which translated into significantly improved 1-year survival, especially in patients identified as being at high risk of death. This finding is consistent with the study of Dunlay et al. where the persistence of severe functional disability was associated with increased risk of subsequent mortality and readmission^52^. These findings suggest that more efforts should be devoted to improve the functional status of severely disabled patients. Recently, the Rehabilitation Therapy in Older Acute Heart Failure Patients (REHAB-HF) trial proved that SFI can be modifiable with a “sustained, targeted, progressive multidomain rehabilitation intervention”^53^, underscoring the importance of a targeted intervention that includes multiple physical-function domains. Consistently with a previous study^54^, our data also suggest that considering 6MWT results after CR might help refining risk assessment.

Our study has some limitations. As with most prognostic studies of HF, this was a retrospective study. However, Rahimi et al. found no evidence that differences in study design (prospective vs. retrospective) are significantly associated with the discriminative ability of a risk model^23^. Our cohort comprised patients with chronic HF admitted to inpatient CR because of a recent hospitalization for HF or declining functional status or clinical conditions, the majority of whom presented with severe functional limitation—that is, a population at relatively high risk in the clinical context of chronic HF. Fifteen percent of the patients died within one year compared with a 1-year mortality of 10% or less reported in previous studies of contemporary cohorts^55,56^. Thus, the SFI/NT-proBNP HF risk score may require recalibration when applied to the general population with chronic HF. As most previously published predictive models for HF^4^, the SFI/NT-proBNP HF risk score, though internally validated, was not tested in an external population. “The lack of external validation makes it difficult to assess how the performance of the model might be generalized to other populations”^57^.

## Conclusions

In conclusion, our data suggest that a simple, 6MWT-derived measure of SFI is a strong predictor of death and provide incremental prognostic information over well-established risk markers in HF, including a NP, and over the MAGGIC score. The multi-parametric point-based SFI/NT-proBNP HF scoring system, which combines demographic, clinical, biochemical, and functional indices, demonstrated good performance in predicting 1-year mortality. Demonstrating improved outcomes depending on a risk score-based clinical management should become a primary target of future prognostic research.

### Supplementary Information

Supplementary Legends.

Supplementary Figure 1.

Supplementary Figure 2.

Supplementary Figure 3.

Supplementary Figure 4.

Supplementary Table 1.

## Data Availability

The dataset analyzed during the current study is not publicly available but is available from the corresponding author upon reasonable request.
