# Supplementary material for: Incremental prognostic value of functional impairment assessed by 6-min walking test for the prediction of mortality in heart failure
Source: Sci Rep. 2024 Feb 7;14:3089. doi: 10.1038/s41598-024-53817-3 (PMC10847418; doi:10.1038/s41598-024-53817-3)
Supplement: Supplementary file 1 — Supplementary Legends. [file 41598_2024_53817_MOESM1_ESM.docx]

Supplementary figure S1. Distribution of the patients across the risk scores (left axix) and predicted probability of 1-year mortality for each score (right axis).

Supplementary figure S2. Prevalence of individual predictors across quintiles of the risk score

Supplementary figure S3. Proportion of patients who achieved an improvement in 6MWD ≥300 m among those with SFI at admission across quintiles of the SFI/NT-proBNP risk score. N, number of patients.

Supplementary figure S4. Cumulative 1-year survival of patients with SFI stratified by improvement in six-minute walking distance to 300 m or more at discharge, across quintiles of the SFI/NT-proBNP risk score. Q denotes quintile, ARR absolute risk reduction, NNT number needed to treat. Number in parentheses are 95% confidence intervals. Because of the low number of patients (N. 33), data for quintile 1 were not reported.
