# Supplementary figures and images for: Incremental prognostic value of functional impairment assessed by 6-min walking test for the prediction of mortality in heart failure
Source: Sci Rep. 2024 Feb 7;14:3089. doi: 10.1038/s41598-024-53817-3 (PMC10847418; doi:10.1038/s41598-024-53817-3)

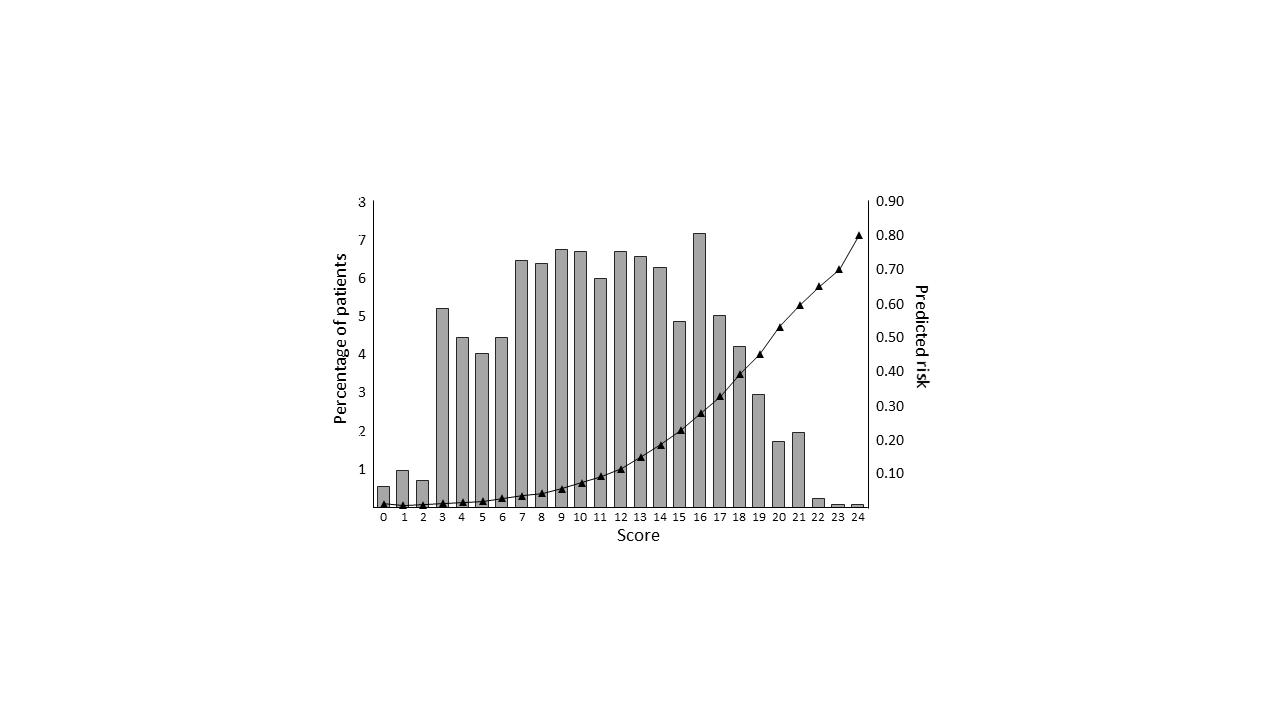

Supplement: Supplementary file 2 — Supplementary Figure 1. [file 41598_2024_53817_MOESM2_ESM.tif]

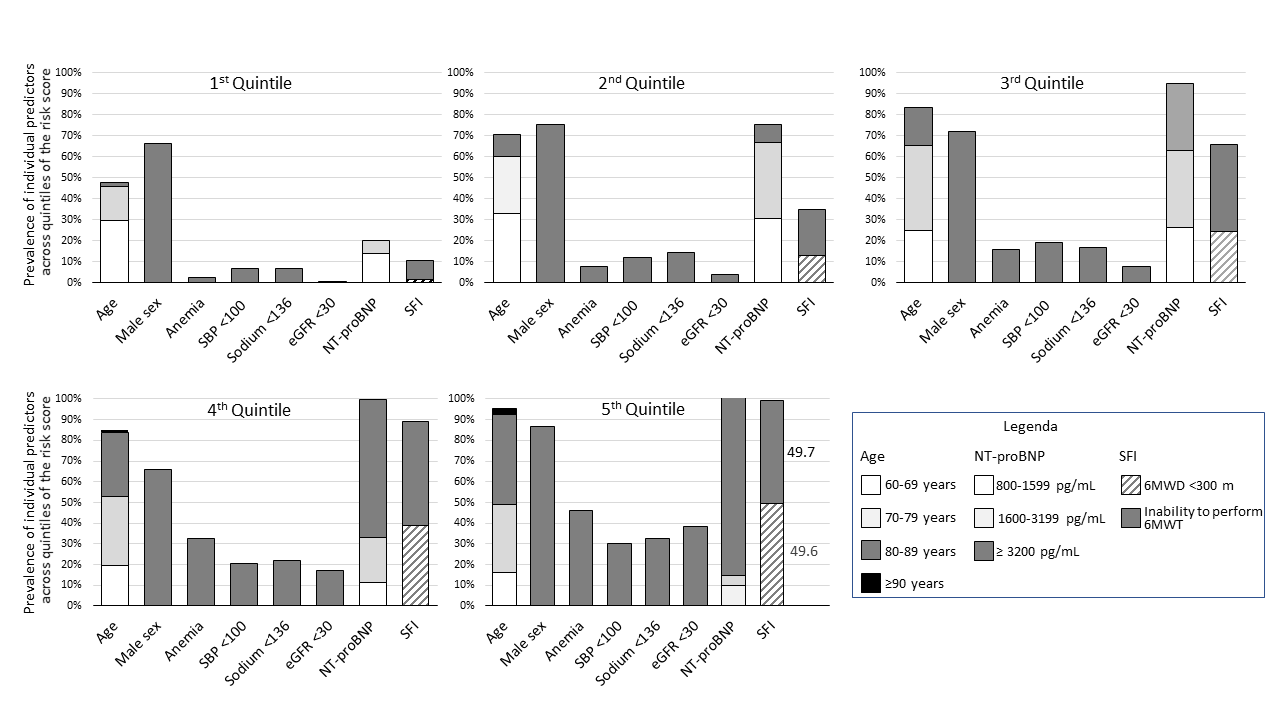

Supplement: Supplementary file 3 — Supplementary Figure 2. [file 41598_2024_53817_MOESM3_ESM.tif]

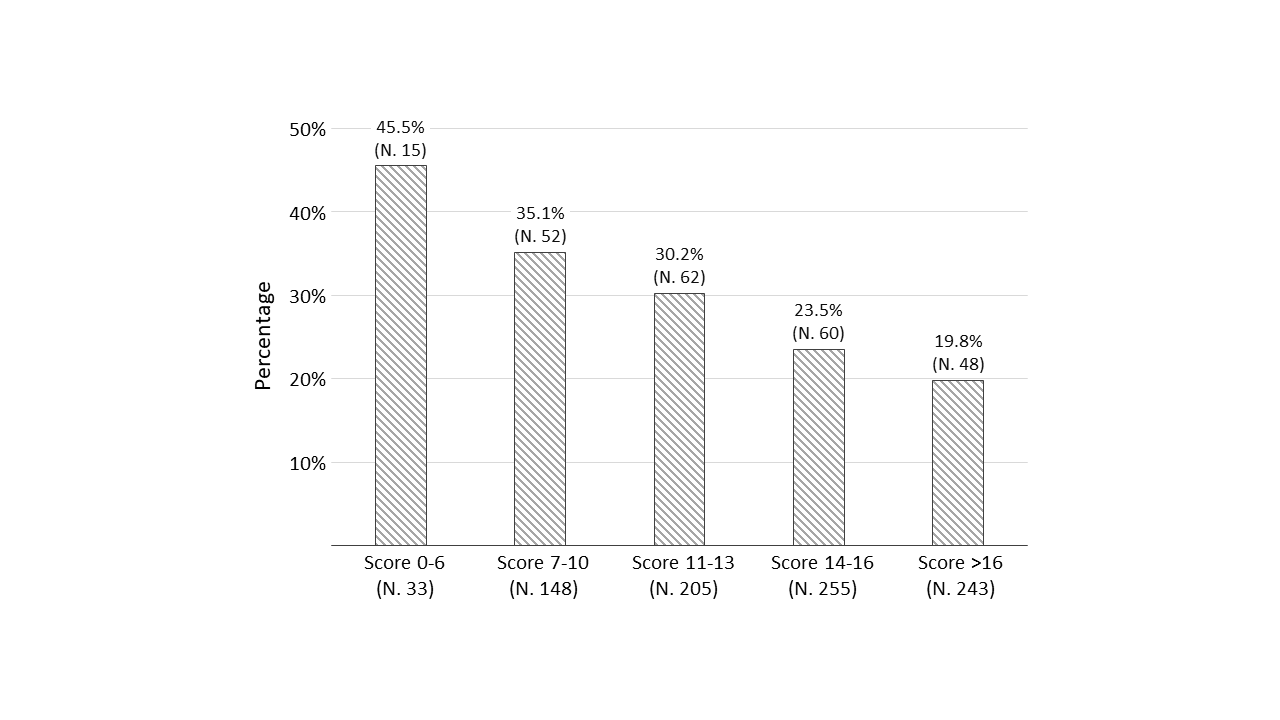

Supplement: Supplementary file 4 — Supplementary Figure 3. [file 41598_2024_53817_MOESM4_ESM.tif]

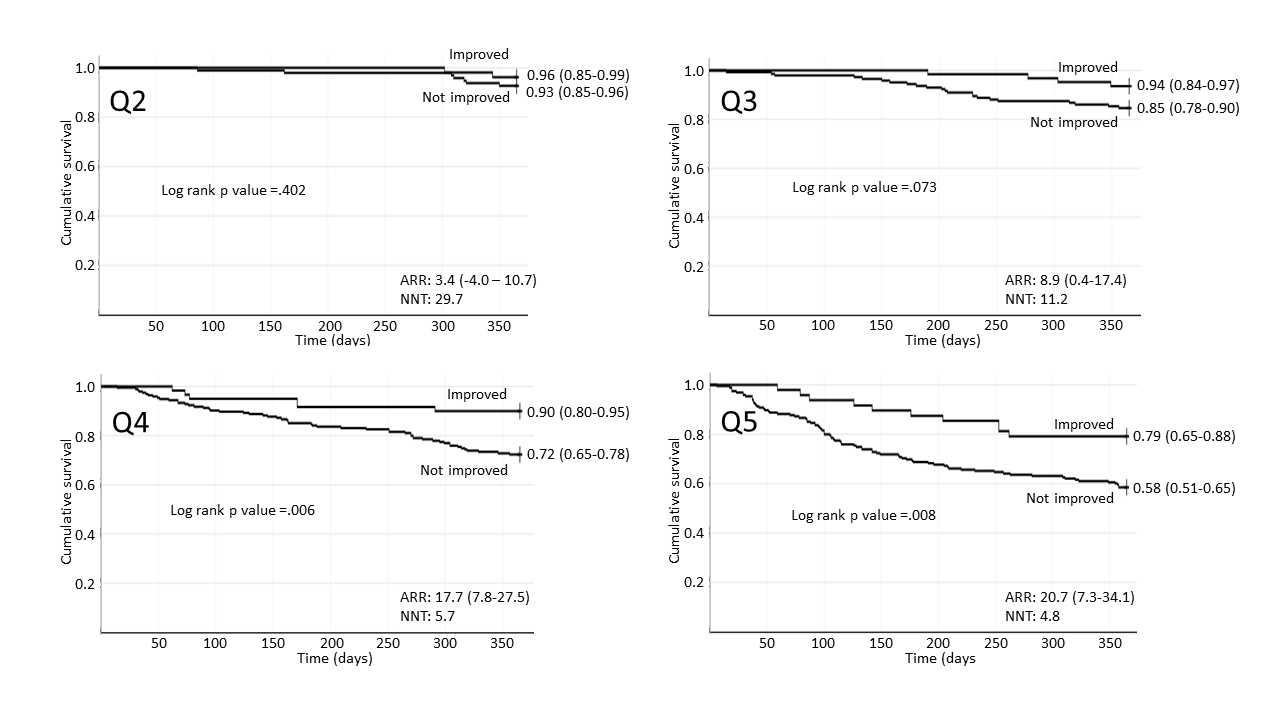

Supplement: Supplementary file 5 — Supplementary Figure 4. [file 41598_2024_53817_MOESM5_ESM.tif]
