# Supplementary material for: Incremental prognostic value of functional impairment assessed by 6-min walking test for the prediction of mortality in heart failure
Source: Sci Rep. 2024 Feb 7;14:3089. doi: 10.1038/s41598-024-53817-3 (PMC10847418; doi:10.1038/s41598-024-53817-3)
Supplement: Supplementary file 6 — Supplementary Table 1. [file 41598_2024_53817_MOESM6_ESM.docx]

Supplemental table 1. Baseline characteristics stratified by functional status.

|  | No SFI  (N. 744) | |  | SFI  (N. 952) | |  |  |
| --- | --- | --- | --- | --- | --- | --- | --- |
| Variables | Number of observations | Mean (SD) or  N (%) |  | Number of observations | Mean (SD) or  N (%) | p value |  |
| **Demographics** |  |  |  |  |  |  |  |
| Age (years), mean (SD) | 744 | 61.7 (11.8) |  | 952 | 72.7 (11.5) | <.001 |  |
| Age >70 years, N (%) | 744 | 183 (24.6) |  | 952 | 611 (64.2) | <.001 |  |
| Females, N (%) | 744 | 114 (15.3) |  | 952 | 342 (35.9) | <.001 |  |
| Body mass index, mean (SD) | 724 | 27.1 (5.5) |  | 931 | 27.1 (6.4) | .998 |  |
| **Etiology** | 659 |  |  | 758 |  |  |  |
| Ischemic heart disease, N (%) |  | 290 (44.0) |  |  | 417 (55.0) |  |  |
| Dilated cardiomyopathy, N (%) |  | 312 (47.3) |  |  | 180 (23.7) |  |  |
| Hypertensive, N (%) |  | 24 (3.6) |  |  | 88 (11.6) |  |  |
| Valvular, N (%) |  | 31 (4.7) |  |  | 62 (8.2) |  |  |
| Others, N (%) |  | 2 (0.3) |  |  | 11 (1.5) |  |  |
| **Comorbidities** |  |  |  |  |  |  |  |
| Obesity (body mass index ≥30), N (%) | 724 | 175 (24.2) |  | 931 | 257 (27.6) | .114 |  |
| Hypertension, N (%) | 744 | 230 (30.9) |  | 952 | 580 (60.9) | <.001 |  |
| Diabetes mellitus, N (%) | 744 | 174 (23.4) |  | 952 | 336 (35.3) | <.001 |  |
| Chronic obstructive pulmonary disease, N (%) | 744 | 106 (14.2) |  | 952 | 240 (25.2) | <.001 |  |
| Moderate-to-severe anemia (hemoglobin <11 g/dL), N (%) | 744 | 61 (8.2) |  | 952 | 259 (27.2) | <.001 |  |
| Atrial fibrillation, N (%) | 744 | 190 (25.5) |  | 952 | 440 (46.2) | <.001 |  |
| **Clinical findings** |  |  |  |  |  |  |  |
| Transferred from acute care hospitals after a hospitalization for HF, N (%) | 744 | 200 (26.9) |  | 952 | 498 (52.3) | <.001 |  |
| NYHA III/IV class, N (%) | 744 | 443 (59.5) |  | 952 | 601 (63.1) | .139 |  |
| ICD, N (%) | 744 | 350 (47.0) |  | 952 | 280 (29.4) | <.001 |  |
| ICD in patients with reduced EF, N (%) | 585 | 313 (53.5) |  | 595 | 251 (42.2) | <.001 |  |
| Systolic blood pressure (mm Hg), mean (SD) | 720 | 110.6 (16.1) |  | 905 | 112.9 (18.1) | <.001 |  |
| Systolic blood pressure <100 mm Hg, N (%) |  | 133 (18.5) |  |  | 154 (17.0) | .444 |  |
| Left ventricular ejection fraction, mean (SD) | 744 | 0.33 (0.11) |  | 952 | 0.38 (0.13) | <.001 |  |
| Left ventricular ejection fraction <0.40, N (%) | 744 | 570 (76.6) |  | 952 | 564 (59.2) | <.001 |  |
| Six-minute walking test |  |  |  |  |  |  |  |
| Inability to walk, N (%) | 744 | 0 (0) | Inability to walk, N (%) | 952 | 400 (42.0) |  |  |
| 6MWD <300 m, N (%) | 744 | 0 (0) | 6MWD <300 m, N (%) | 952 | 552 (58.0) |  |  |
| Six-minute walking distance in patients able to perform a 6MWT (meters), mean (SD) | 744 | 390 (66) |  | 552 | 199 (68) | <.001 |  |
| **Laboratory findings** |  |  |  |  |  |  |  |
| Hemoglobin (g/dL), mean (SD) | 744 | 13.5 (1.7) |  | 952 | 12.3 (2.0) | <.001 |  |
| Creatinine (mg/dL), (mean (SD) | 744 | 1.25 (0.41) |  | 952 | 1.50 (0.66) | <.001 |  |
| eGFR (mL/min/1.73 m^2^), mean (SD) | 744 | 63.2 (21.5) |  | 952 | 51.0 (24.1) | <.001 |  |
| eGFR <60 mL/min/1.73 m^2^, N (%) | 744 | 334 (44.9) |  | 952 | 662 (69.5) | <.001 |  |
| 45-59 mL/min/1.73 m^2^, N (%) |  | 182 (24.5) |  |  | 229 (24.1) | <.001 |  |
| 30-44 mL/min/1.73 m^2^, N (%) |  | 123 (16.5) |  |  | 261 (27.4) |  |  |
| <30 mL/min/1.73 m^2^, (N%) |  | 29 (3.9) |  |  | 172 (18.1) |  |  |
| Sodium <136 mEq/L, N (%) | 744 | 175 (23.5) |  | 952 | 127 (13.3) | <.001 |  |
| NT-proBNP (pg/mL), median (IQR) | 744 | 1238 (559.2776) |  | 952 | 3071 (1174-6143) | <.001 |  |
| <800 pg/mL, N (%) |  | 255 (34.3) |  |  | 147 (15.4) | <.001 | |
| 800-1599 pg/mL, N (%) |  | 154 (20.7) |  |  | 154 /16.2) |  |  |
| 1600-3199 pg/mL, N (%) |  | 188 (25.3) |  |  | 194 (20.4) |  |  |
| ≥3200 pg/mL, N (%) |  | 147 (19.8) |  |  | 457 (48.0) |  |  |

Abbreviations. eGFR denotes estimated glomerular filtration rate, ICD implantable cardioverter defibrillator, NYHA New York Heart Association, N number of patients, SD standard deviation, 6MWT six-minute walking test.
